# The effect of maternal and child factors on stunting, wasting and underweight among preschool children in Northern Ghana

**DOI:** 10.1186/s40795-017-0154-2

**Published:** 2017-04-04

**Authors:** Zakari Ali, Mahama Saaka, Abdul-Ganiyu Adams, Stephen K. Kamwininaang, Abdul-Razak Abizari

**Affiliations:** Department of Community Nutrition, School of Allied Health Sciences, University for Development Studies, P O Box 1883, Tamale, Ghana

**Keywords:** Preschool children, Stunting, Wasting, Underweight, Child factors, Dietary diversity, Ghana

## Abstract

**Background:**

Undernutrition among preschool children in Northern region is the highest in Ghana. However, there is scarcity of data on the factors that determine undernutrition in these children. This study investigated the effect of maternal and child factors on undernutrition among preschool children in Northern Ghana.

**Methods:**

This study was a community based analytical cross-sectional survey on a sample of 425 mother- child pairs drawn from 25 clusters. A semi- structured questionnaire was used to collect data on maternal and child socio-demographic characteristics, feeding practices and anthropometry. Anthropometric indices of Height-for-age Z-scores (HAZ), Weight-for-height Z-scores (WHZ) and Weight-for – age Z-scores (WAZ) were used to classify child stunting, wasting and underweight respectively. Bivariate and multivariate analyses were performed to determine associations between explanatory variables and undernutrition.

**Results:**

The prevalence of stunting, wasting and underweight were 28.2, 9.9 and 19.3% respectively. Multiple logistic regression analysis showed that, the odds of stunting was higher among male children [AOR = 1.99; 95% CI (1.26–3.13); *p* = 0.003], children of mothers less than 150 cm in height [AOR = 3.87; 95% CI (1.34–11.20); *p* = 0.01], mothers 155–159 cm tall [AOR = 2.21; 95% CI (1.34–3.66); *p* = 0.002], and older children aged 12–23 months [AOR 9.81; 95% CI (2.85–33.76); *p* < 0.001]. Wasting was significantly higher among male children [AOR = 2.40; 95% CI (1.189–4.844); *p* = 0.015], consumption of less than four food groups [AOR = 3.733; 95% CI (1.889–7.376); *p* < 0.001] and among children of underweight mothers [AOR = 3.897; 95% CI (1.404–10.820); *p* = 0.009]. Male children [AOR = 2.685; 95% CI (1.205–5.98); *p* = 0.016] and having low birth weight [AOR = 3.778; 95% CI (1.440–9.911); *p* < 0.001] were associated with higher odds of underweight in children.

**Conclusion:**

Maternal height associated negatively with stunting but not wasting. Factors that affect low height –for-age z-score (HAZ) may not necessarily be the same as stunting. Infant and child feeding practices as measured by dietary diversity score associated positively with weight-for-height Z-scores than length-for-age Z-scores of young children. Surprisingly, consumption of some specific food groups including, animal source foods, legumes, staples and eggs were associated with lower HAZ but with increased likelihood of higher WHZ among children 6–59 months.

**Electronic supplementary material:**

The online version of this article (doi:10.1186/s40795-017-0154-2) contains supplementary material, which is available to authorized users.

## Background

Malnutrition is of public health importance in developing countries and is responsible for over half of child deaths each year from preventable causes [1]. Stunting is defined as height-for-age below minus two standard deviations (−2SD) from the median of WHO reference population [2] and considered an indicator of long-term nutritional inadequacy [3]. Stunting in children, especially in the first 2 years of life has long term effects on adult height, income, school attainment [4], and a predisposition to adult chronic diseases [5].

Although over the past decades child stunting has decreased worldwide, it has remained high in Sub-Saharan Africa and Asia and afflicts an estimated 165 million children globally [6]. In Ghana, there has been a decline in stunting prevalence but still remains high in children under 5 years. The Ghana demographic and health survey (GDHS) reports indicate a decrease in child stunting from 28% in 2008 [2] to 19% in 2014 [7]. Though this is an encouraging reduction nationally, there are wide disparities among the regions [8, 9], with most southern regions having lower prevalence and the northern regions with higher prevalence [9]. Stunting in Northern region is the highest in Ghana with a prevalence of 33% among preschool children [7].

Despite the high prevalence of stunting among children, its determinants are poorly established and understood [10] and limited studies have been undertaken especially in the Northern region to identify the determinants of malnutrition among children [11]. As Ghana strives to attain the Sustainable Development Goals (SDGs) by the year 2030, ensuring nutritional well-being is key [8]. Government and non-governmental organizations in response to the high prevalence of stunting among children in the Northern region have initiated some interventions to decrease it [9]. For these interventions to have the most impact on stunting, data on the predictors and the strong determinants of stunting affecting these children is critical. Moreover, data is scarce especially in the Northern region on the determinants of wasting and underweight in children. This study therefore investigated maternal and child factors that are associated with stunting, wasting and underweight among preschool children in the Central Gonja district of Northern Ghana.

## Methods

### Study area

The study was conducted in the Central Gonja District which is located at the south-western part of the Northern Region of Ghana with its administrative capital at Buipe. The District covers approximately 7555 km^2^ land area which represent 11.0% of the total land area of the region and shares boundary with the southern parts of Ghana. The district is divided into five sub- districts for administrative purposes with a total population of 87,877 with 49.9% as males and 50.1% as females. The population of the district is largely youthful with children aged 0–4 years forming the largest part representing 17.6% of the total population. The main economic activity of the people is agriculture (74.2%) involving crop production, livestock and fish farming. Some of the crops cultivated are maize, sorghum, millet, groundnut, cowpea, soy beans, yam, rice, as well as cassava. Fishing and livestock are considered supplementary activities to crop farming [12].

### Study design, population and sampling

A community-based analytical cross-sectional design was used in this study. The target population was children under five years and their mothers/primary caregivers. In households with more than one eligible child, one child was chosen at random to participate in the study. An eligible child and its mother were all present at the point of data collection for inclusion.

A sample size of 425 was determined on the assumption that the prevalence of stunting in the study district was unknown and so 50% was assumed with 5% marginal error and 95% confidence interval (CI) and a none response rate of 10%.

The study used a multi-stage cluster sampling procedure which involved selecting communities and households. To make valid conclusions in a community- based cluster study, a minimum of 25 clusters is usually required [13, 14]. This study used this minimum number of clusters by randomly choosing 25 communities from a list of communities from four sub-districts across the Central Gonja district using Excel generated random numbers. The primary sampling units in selected clusters was households. With a required sample size of 425 and 25 clusters, the minimum sample required for each cluster was 17 households. Systematic sampling technique was used to select the required number of households in each cluster. A list of all households with an eligible child (a child aged 6–59 months) in a selected cluster was compiled and numbered in ascending order. The sampling interval for the cluster was then determined by dividing the total number of households 17. A number within the sampling interval was randomly selected to get the first household to visit. Subsequently, households to visit were identified by adding the sampling interval to the number selected. This was done until the 17 household required from a cluster was obtained. In households with more than one eligible child mother pairs, one was selected for interview using simple random sampling technique.

### Data collection procedures

Face-to-face interviews were conducted in homes using pre-tested and validated structured questionnaires to collect representative data on socio-demographic characteristics, illness history, nutritional status, health and nutrition behaviors of mothers/caretakers. Information on the economic well-being of families (e.g. household wealth index) was also collected (see Additional fie 1 for study questionnaire).

### Variables measured

Both potential proximal and distal determinants of stunting were investigated. The proximal determinants relate to biological functions of both mother and child or to specific maternal practices that influence food in-take, health, and caregiving. These included child’s gender, age in months, birth weight, birth interval, breastfeeding status, dietary intake, diarrhoea and fever in the last two weeks, mother’s nutritional status, mother’s age, maternal health seeking behaviors such as prenatal and postnatal care for mothers, and caring practices for children. The distal determinants are the resources necessary for achieving adequate food security, care, and a healthy environment. The distal determinants assessed included household socioeconomic status, maternal education, access to safe water and access to sanitary toilet facilities.

Briefly, a description of main independent variables is as follows:

### Nutritional status assessment

#### Age

The exact age of the child was recorded in months, based on date of birth information contained in child health records booklets, birth certificates and baptismal cards.

#### Weight

The weight of children was assessed with Seca Electronic UNISCALE (SECA 890). Weight was measured to the nearest gram. Weighing scale was calibrated to zero before taking every measurement. The children were weighed with minimal clothing, without shoes and with minimal movement on the scale. Tare weighing was performed for children who could not stand well on the scale to take a meaningful measure.

#### Length

The length measurements of children less than two years of age (i.e. up to and including 23 months) were taken following WHO standard procedures. A specialized wooden device (that is, an infantometer) was used to take the measurement in a supine position. This was done by placing the child on its back between the slanting sides with the head placed gently against the fixed top end. The knees were gently pushed down by a helper while the foot-piece is moved toward the child until it presses softly against the soles of the child’s feet and the feet are at right angles to the legs. The length was then read to the nearest 0.1 cm. For children who were more than two years, height was measured using the infantometer in standing position.

Anthropometric data were then transformed into Z-scores for height-for-age Z-score (HAZ), weight-for-length Z-score (WLZ) and weight-for-age Z-score (WAZ). Categorical variables were stunting, wasting and underweight which reflect HAZ, WAZ and WLZ below −2 standard deviations below the population median.

#### Body Mass Index (BMI)

BMI is a simple index of weight-to-height commonly used to classify underweight, overweight and obesity in adults. It is defined as the weight in kilograms divided by the square of the height in metres (kg/m^2^). Weight of mothers was measured with a Seca electronic weighing scale to the nearest 0.1 kg. Height of mothers was measured with a Seca microtoise to the nearest 0.1 cm.

#### Bilateral edema

This was diagnosed by placing both thumbs on the upper side of the feet and applying pressure for about three seconds. Edema was considered to be present if a skin depression remained on both feet after the pressure was released.

### Assessment of dietary intake

Overall dietary quality was assessed using the dietary diversity score. The WHO validated 7-item food groups frequency questionnaire (FFQ) was used to quantify child dietary intake [15]. Food that was fed to the children was assessed using a structured 24-h food frequency questionnaire. Mothers were asked to recall the number of times, in the past 24 h, a child had received anything to eat, aside from breast-milk, including meals and snacks. The dietary diversity score therefore ranged from 0 to 7 with minimum of 0 if none of the food groups is consumed to 7 if all the food groups are consumed.

The WHO defined minimum dietary diversity as the proportion of children aged 6–23 months who received foods from at least four out of seven food groups [15, 16]. The seven food groups used in defining children’s minimum dietary diversity indicator are:

(i) grains, roots and tubers; (ii) legumes and nuts; (iii) dairy products; (iv) flesh foods (meats/fish/poultry) (v) eggs (vi) vitamin A rich fruits and vegetables; and (vii) other fruits and vegetables.

WHO Infant and young child feeding (IYCF) indicators [minimum dietary diversity (MDD), minimum meal frequency (MMF), minimum acceptable diet (MAD)] were the main dietary intake indicators for children. These indicators were measured by recall of food and liquid consumption during the previous day or night preceding survey as per WHO/UNICEF food frequency questionnaire (FFQ) [17]. Minimum meal frequency is the proportion of children who received complementary foods at least the minimum recommended number of times in the last 24 h. A child is judged to have taken ‘adequate number of meals if he/she received at least the minimum frequency for appropriate complementary feeding (that is, 2 times for 6–8 months and 3 times for 9–11 months, 3 times for children aged 12–23 months) in last 24 h. For non-breastfed children, the minimum meal frequency is 4.

### Assessment of socio-economic status

A household wealth index based on household assets and housing quality was used as a proxy indicator for socio-economic status (SES) of households.

Absolute household wealth index was calculated from information collected on housing quality (floor, walls, and roof material), source of drinking water, type of toilet facility, the presence of electricity, type of cooking fuel, and ownership of modern household durable goods and livestock (e.g. bicycle, television, radio, motorcycle, sewing machine, telephone, cars, refrigerator, mattress, bed, computer and mobile phone) [18–21]. These facilities or durable goods are often regarded as modern goods that have been shown to reflect household wealth. A household of zero index score for example means that household had not a single modern good. The scores were thus added up to give the proxy household wealth index. The median score of 7 was used as the cut-off point to define low and high household wealth index.

### Data quality assurance

A number of measures were used to ensure that accurate and reliable data were collected and analyzed. These included extensive practical training of data enumerators, pre-testing of data collection tools and extensive monitoring of the field teams. Field supervisors provided on-the-spot assistance to interviewers. Data were checked for completeness and consistency by field supervisors in the field and during data entry in order to ensure good quality.

### Statistical analysis

Data analyses was performed with PASW/SPSS, version 21.0 for Windows using procedures in SPSS complex samples module for Windows. Design weights were added to perform weighted analysis. This module of SPSS takes into account the complex nature of the cluster sample design.

The Z- scores for weight-for-age (WAZ), weight-for- height (WHZ), height-for-age (HAZ) and prevalence underweight, wasting and stunting were calculated using the 2006 World Health Organization (WHO) growth standards.

WHO Anthro software was used to convert height, weight and age measurements to Height- for- age Z- scores (HAZ), weight- for –height Z- scores (WHZ) and weight-for – age (WAZ) which were used to classify stunting, wasting and underweight respectively. Child undernutrition was defined as Z- scores below −2 standard deviations below the median of the reference population.

Bivariate and multivariate analyses were both performed to identify the determinants of stunting, wasting and underweight. Chi-square (*χ* ^*2*^) tests were performed to identify the predictors of stunting, wasting and underweight significant at *p* < 0.05. ANOVA was used to compare mean anthropometric Z-scores and selected predictors.

The association between undernutrition and the independent variables was determined using binary logistic regression modeling. This test statistic was used because stunting, wasting and underweight were coded into two categories (that is: stunted and normal, wasted and normal, and underweight and normal). All of the potential predictors of stunting, wasting and underweight that were significant at *p* < 0.05 in bivariate analysis using Chi-square (*χ* ^*2*^) tests were included in the regression modeling.

The association between selected factors and HAZ was determined using multiple linear regression modeling. This test statistic was used because the dependent factor (HAZ) is a continuous variable.

Before testing for associations between independent variables and the dependent outcomes, the data were cleaned and outliers removed. Z-scores which were outside the WHO flags: WHZ −5 to 5; HAZ −6 to 6; and WAZ −6 to 5 were excluded from the data set.

### Ethics consideration

Permission to carry out this study was sought from the district health directorate and the study protocol was approved by the School of Allied Health Sciences, University for Development Studies, Ghana. Informed consent was verbally obtained from participant mothers after needed information and procedures were explained. Participation was voluntary and participants signed or thumb printed a statement of an informed consent before being interviewed.

## Results

### Socio-demographic characteristics of the sample

The mean age ± SD of mothers was 30.3 ± 6.0 years with the majority (75.3%) in the 18–34 age groups. The majorities of the respondents had no formal education (78.1%), were married (91.3%), practiced Islamic religion (93.4%) and mostly from the Gonja ethnic group (53.6%). Sixty-three percent of mothers were from low wealth households.

Children had mean age ± SD of 25.14 ± 13.6 months; male children (50.8%) were slightly more than the female children (49.2%) with most of the children found in the age group 12–23 months (32.5%). Majority of the children had adequate birth weight ≥ 2.5 and a mean birth weight of 2.97 ± 0.49 (Table 1).

**Table 1:** Socio- demographic characteristics of the study population

Characteristics	Mean (± SD)	Frequency (n)	Percentage (%)
Mean age (years)	30.26 ± 6.02		
Age groups (years)			
18–34		320	75.3
At least 35		105	24.7
Education			
None		332	78.1
Low (primary/junior high school)		79	18.6
High (at least sec school)		14	3.6
Marital status			
Married		388	91.3
Currently unmarried		37	8.7
Ethnicity			
Gonja		228	53.6
Dagomba		115	27.1
Others		82	19.3
Religion			
Islam		397	93.4
Other religions		28	6.6
Household wealth			
Low		268	63.1
High		157	36.9
Child characteristic			
Mean age (months)	25.14 ± 13.56		
Age groups (months)			
6–11		72	16.9
12–23		138	32.5
24–35		108	25.4
36–47		70	16.5
48–59		37	8.7
Sex			
Male		216	50.8
Female		209	49.2
Mean birth weight (kg)	2.97 ± 0.49		
Birth weight (kg)			
< 2.5		23	11.9
≥ 2.5		170	88.1

### Nutritional status and dietary intake of children under-five years

The nutritional status and dietary practices of children are presented in Table 2. The negative z-scores for the entire study population indicate that the children in the study sample are less well-nourished relative to the WHO standard population. The prevalence of chronic, acute and underweight was 28.2, 9.9 and 19.3% respectively. The mean dietary diversity score (DDS) was 3.58 ± 1.58 and less than 50.0% of the children met the minimum dietary diversity (≥4 food groups) and minimum acceptable diet. Children who met the acceptable diet and also started complementary feeding at six months were considered to have appropriate complementary feeding. Therefore, the overall appropriate complementary prevalence was only 15.5%.

**Table 2:** Nutritional status and dietary intake of children under-five years

Characteristics	Mean ± SD	Frequency (n)	Percentage (%)
Nutritional status			
Height-for age-z-score (HAZ)	−1.33 ± 1.2		
Weight-for-height-z-score (WHZ)	−0.64 ± 1.1		
Weight-for-age-z-score (WAZ)	−1.18 ± 1.1		
Stunted (HAZ < −2)		120	28.2
Wasted (WHZ < −2)		42	9.9
Underweight (WAZ < −2)		82	19.3
Overweight (WHZ > 2)		8	1.9
Feeding practices			
Currently breastfeeding		217	51.1
Timely initiation of breast feeding within one hour		211	49.6
Introduction of complementary foods at 6 months		162	38.1
Received colostrum		375	88.2
*Minimum meal frequency (children aged 6–23 months)		141	64.7
*Minimum dietary diversity (≥4 food groups)		100	45.9
*Minimum Acceptable diet (children aged 6–23 months)		91	41.7

*Sample size (n) for children 6–23 months = 218

### Predictors of mean HAZ and WHZ among children aged 6–59 months

Bivariate analysis of the predictors of mean height- for- age Z-score (HAZ) and weight-for-height Z-scores (WHZ) are shown in Tables 3 and 4. Infant and young child feeding (IYCF) practices indicators better explain weight-for-height Z-scores (WHZ) than height-for-age Z-scores (HAZ).

**Table 3:** Relationship between mean height –for-age z-score (HAZ) and selected variables among children aged 6–59 months

Indicator	N	Mean HAZ	Std. Deviation	95% Confidence Interval for Mean		*p* value
				Lower Bound	Upper Bound	
Child currently breastfeeding?						
No	208	−1.4115	1.17570	−1.5722	−1.2508	0.2
Yes	217	−1.2504	1.25697	−1.4186	−1.0822	
Age of child (months)						
6–8	46	-.1770	1.44827	-.6070	.2531	<0.001
9–11	30	-.6190	1.42657	−1.1517	-.0863	
12–23	142	−1.5582	1.15160	−1.7493	−1.3672	
24–59	207	−1.5311	.97958	−1.6653	−1.3969	
Gender of child						
Male	216	−1.4953	1.32478	−1.6730	−1.3177	0.004
Female	209	−1.1576	1.07576	−1.3043	−1.0109	
Timing of first complementary food at 6 months						
No	263	−1.4068	1.11418	−1.5421	−1.2716	0.09
Yes	162	−1.2032	1.36677	−1.4153	-.9911	
Minimum meal frequency						
No	190	−1.2549	1.27073	−1.4367	−1.0730	0.3
Yes	235	−1.3893	1.17502	−1.5403	−1.2383	
Minimum dietary diversity						
Less than 4 groups	154	−1.0892	1.37680	−1.3083	-.8700	0.002
At least 4 groups	271	−1.4656	1.09919	−1.5971	−1.3342	
Minimum acceptable diet						
No	251	−1.2239	1.32109	−1.3882	−1.0597	0.03
Yes	174	−1.4811	1.03980	−1.6367	−1.3255	
Classification of household wealth index						
Low	268	−1.4153	1.12989	−1.5512	−1.2794	0.06
High	157	−1.1823	1.34907	−1.3950	-.9696	
Maternal height (cm)						
< 150	17	−1.8812	1.04163	−2.4167	−1.3456	0.004
150–154	57	−1.4512	1.41418	−1.8265	−1.0760	
155–159	129	−1.5319	1.07278	−1.7188	−1.3450	
At least 160	222	−1.1379	1.22944	−1.3005	-.9753	
Classification of birth weight						
Normal (≥2.5 kg)	170	−1.1429	1.25864	−1.3335	-.9524	<0.001
Low birth weight (<2.5 kg)	23	−2.1230	1.06457	−2.5834	−1.6627	
Consumption of staples in the past 24 h prior to study						
No	43	-.7381	1.15345	−1.0931	-.3832	<0.001
Yes	382	−1.3958	1.20973	−1.5175	−1.2741	
Consumption of animal source foods in the past 24 h prior to study						
No	94	-.9859	1.31444	−1.2551	-.7166	0.002
Yes	331	−1.4267	1.17454	−1.5537	−1.2997	
Consumption of legumes foods in the past 24 h prior to study						
No	211	−1.1699	1.30031	−1.3463	-.9934	0.007
Yes	214	−1.4864	1.11425	−1.6365	−1.3362

**Table 4:** Relationship between mean weight –for-height z-score (WHZ) and selected variables among children aged 6–59 months

Indicator	N	Mean WHZ	Std. Deviation	95% Confidence Interval for Mean		*p* value
				Lower Bound	Upper Bound	
Child currently breastfeeding?						<0.001
No	208	−0.41	0.94	−0.54	−0.28	
Yes	217	−0.87	1.20	−1.03	−0.71	
Age of child (months)						<0.001
6–8	46	−0.82	1.02	−1.12	−0.52	
9–11	30	−1.32	1.27	−1.79	−0.85	
12–23	142	−0.76	1.197	−0.96	−0.57	
24–59	207	−0.43	0.98	−0.56	−0.29	
Gender of child						0.06
Male	216	−0.74	1.19	−0.90	−0.59	
Female	209	−0.54	1.01	−0.68	−0.40	
Timing of first complementary food at 6 months						0.8
No	263	−0.64	1.059	−0.76	−0.51	
Yes	162	−0.66	1.18	−0.84	−0.48	
Minimum meal frequency						0.7
No	190	−0.62	1.09	−0.77	−0.46	
Yes	235	−0.67	1.12	−0.81	−0.52	
Minimum dietary diversity						<0.001
Less than 4 groups	154	−0.98	1.17	−1.17	−0.79	
At least 4 groups	271	−0.45	1.02	−0.58	−0.33	
Minimum acceptable diet						0.13
No	251	−0.71	1.13	−0.85	−0.57	
Yes	174	−0.55	1.06	−0.71	−0.39	
Classification of household wealth index						0.3
Low	268	−0.60	1.07	−0.73	−0.47	
High	157	−0.72	1.16	−0.91	−0.54	
Maternal height (cm)						0.2
< 150	17	−0.56	1.12	−1.14	0.02	
150–154	57	−0.91	1.20	−1.23	−0.60	
155–159	129	−0.54	1.08	−0.72	−0.35	
At least 160	222	−0.65	1.09	−0.79	−0.50	
Classification of birth weight						0.1
Normal (≥2.5 kg)	170	−0.56	1.03	−0.71	−0.40	
Low birth weight (<2.5 kg)	23	−0.90	1.06	−1.36	−0.44	
Consumption of staples in the past 24 h prior to study						0.1
No	43	−0.93	1.26	−1.32	−0.54	
Yes	382	−0.61	1.08	−0.72	−0.50	
Consumption of animal source foods in the past 24 h prior to study						<0.001
No	94	−1.01	1.20	−1.25	−0.76	
Yes	331	−0.54	1.06	−0.66	−0.43	
Consumption of legumes foods in the past 24 h prior to study						<0.001
No	211	−0.84	1.08	−0.99	−0.69	
Yes	214	−0.45	1.10	−0.60	−0.30	

Whereas, HAZ associated negatively with minimum dietary diversity as well as the consumption of some specific food groups including, animal source foods and legumes, WHZ associated positively with these variables. Other variables that were tested but were not in any way associated with HAZ and WHZ were the educational level of the mother, initiation and frequency of ANC attendance.

### Predictors of stunting among children

A host of explanatory variables were tested for their association with child stunting. The variables tested included age and sex of child, type of residence, current breast feeding status, diarrhoeal infection, utilization of antenatal care services, birth interval, parity, birth weight, maternal height, maternal educational level, household wealth index, source of drinking water, type of toilet facility, bottle feeding, minimum dietary diversity score and number of under five children in household.

Bivariate analysis revealed that, stunting was significantly higher among male children than their female counterparts. Stunting was less prevalent among children whose mothers attended antenatal care (ANC) services for at least 4 times during pregnancy. In addition, stunting was significantly higher among children who were born low birth weight (<2.5 kg) compared to children with adequate birth weight. The prevalence of stunting decreased as maternal height increased, and children who lived in households without a toilet facility were also more likely to be stunted (Table 5).

**Table 5:** Bivariate analysis of predictors of chronic malnutrition among children aged 6–59 months

Characteristic	N	Classification of stunting		*p* value
		Normal n (%)	Stunted n (%)	
Age of child (months)				<0.001
6–11	72	65 (90.3)	7 (9.7)	
12–23	138	88 (63.8)	50 (36.2)	
24–35	108	69 (63.9)	39 (36.1)	
36–47	70	52 (74.3)	18 (25.7)	
48–59	37	31 (83.8)	6 (16.2)	
Classification of ANC visits				0.03
< 4	147	96 (65.3)	51 (34.7)	
≥ 4	278	208 (74.8)	70 (25.2)	
Birth weight classification				<0.001
< 2.5 Kg	23	9 (39.1)	14 (60.9)	
≥ 2.5 Kg	170	129 (75.9)	41 (24.1)	
Gender of child				0.005
Male	216	142 (65.7)	74 (34.3)	
Female	209	163 (78.0)	46 (22.0)	
Type of toilet facility				0.03
No facility (open defecation)	377	264 (70.0)	113 (30.0)	
Pit latrine/flush	48	41 (85.4)	7 (14.6)	
Maternal height (cm)				0.01
< 150	17	9 (52.9)	8 (47.1)	
150–154	57	40 (70.2)	17 (29.8)	
155–159	129	83 (64.3)	46 (35.7)	
≥ 160	222	173 (77.9)	49 (22.1)	

### Predictors of stunting and mean HAZ

Factors that affect low height –for-age z-score (HAZ) may not necessarily be the same as stunting (Tables 6 and 7).

**Table 6:** Multivariate analysis of the determinants of child stunting

	*p* value	AOR	95% C.I. for AOR	
			Lower	Upper
Male child	0.003	1.99	1.26	3.13
Maternal height (Reference: At least 160 cm)	0.004			
<150 cm	0.01	3.87	1.34	11.20
150–154 cm	0.24	1.50	0.76	2.97
155–159 cm	0.002	2.21	1.34	3.66
Age of child (Reference: 6–8 months)	0.001			
9–11	0.14	3.24	0.69	15.10
12–23	<0.001	9.81	2.85	33.76
24–59	0.005	5.87	1.73	19.94
Constant	0.000	0.031

**Table 7:** Predictors of Mean HAZ (Multiple Linear Regression)

Model	Standardized Coefficients	t	*p* value	95.0% Confidence Interval for β	
	Beta			Lower Bound	Upper Bound
(Constant)		−2.861	0.004	−1.66	−0.31
Female child	0.113	2.590	0.010	0.07	0.48
Prevalence of wasting	−0.113	−2.540	0.011	−0.82	−0.11
Currently breastfeeding?	−0.256	−4.413	<0.001	−0.90	−0.35
Classification of maternal height	0.172	3.991	<0.001	0.13	0.37
Protected drinking water	0.100	2.328	0.020	0.04	0.45
Timely introduction of complementary foods	0.097	2.235	0.026	0.03	0.46
Age groups of children	−0.521	−9.096	<0.001	−0.79	−0.51

Multiple logistic regression analysis showed that, age of child, low maternal height and gender of the child were the three significant consistent predictors of stunting. This set of variables accounted for 13.8% of the variability of stunting (Nagelkerke R Square = 0.138).

Male children were 1.9 times more likely [AOR = 1.99; 95% CI (1.26–3.13); *p* = 0.003] of being stunted compared to female children.

Compared to mothers with heights at least 160 cm, others who were less than 150 cm in height were about 3.9 times more likely to have a stunted child [AOR = 3.87; 95% CI (1.34–11.20); *p* = 0.01] and mothers 155–159 cm tall also had significantly higher odds of having a stunted child [AOR = 2.21; 95% CI (1.34–3.66); *p* = 0.002]. Older children aged 12–23 months were 9.8 times greater odds [AOR 9.81; 95% CI (2.85–33.76); *p* < 0.001] of being stunted, compared to children aged 6–8 months.

As age increases (β = −0.521, *p* < 0.001), HAZ score decreases (Table 7). Having introduced complementary food at 6 months (β = 0.097, *p* = 0.026) was associated with a higher HAZ score whereas continued breastfeeding (β = −0.256, *p* < 0.001) was associated with a lower HAZ score. Higher maternal height (β = 0.172, *p* < 0.001) and improved water sources (β = 0.100, *p* = 0.020) are also associated with a higher HAZ score. Wasted children (β = −0.113, *p* = 0.011) were associated with lower values of HAZ. The adjusted R-square for the set of predictors of HAZ was 0.216 (21.6%).

### Predictors of wasting among children

Table 8 presents the bivariate analysis of predictors of wasting in children. Acute malnutrition was associated with sex of child, child age group, birth weight, minimum dietary diversity, consumption of vitamin A rich fruits and vegetables, consumption of flesh foods, and the presence of other children under five years in the household and maternal body mass index. Younger children were more likely to experience wasting than their older counterparts. Children who consumed vitamin A rich fruits and vegetables, and flesh foods (meat, chicken and fish) were less likely to experience acute malnutrition. The presence of other children under six months of age was also associated with acute malnutrition in children.

**Table 8:** Bivariate analysis of predictors of wasting among children

Characteristic	N	Classification of wasting		*p* value
		Normal n (%)	Wasted n (%)	
Sex				0.013
Male	216	187 (86.6)	29 (13.4)	
Female	209	196 (93.8)	13 (6.2)	
Age group of child (months)				0.001
6–11	72	59 (81.9)	13 (18.1)	
12–23	138	117 (84.8)	21 (15.2)	
24–35	108	102 (94.4)	6 (5.6)	
36–47	70	69 (98.6)	1 (1.4)	
48–59	37	36 (97.3)	1 (2.7)	
Birth weight				0.036
< 2.5 Kg	23	18 (78.3)	5 (21.7)	
≥ 2.5 Kg	170	158 (92.9)	12 (7.1)	
BMI of mother				0.015
Underweight	27	20 (74.1)	7 (25.9)	
Normal	306	279 (91.2)	27 (8.8)	
Overweight	92	84 (91.3)	8 (8.7)	
Other children under 3 years in household				0.030
Yes	341	302 (88.6)	39 (11.4)	
No	84	81 (96.4)	3 (3.6)	
Minimum dietary diversity				<0.001
Yes	271	256 (94.5)	15 (5.5)	
No	154	127 (82.5)	27 (17.5)	
Consumption of vitamin A rich fruits and vegetables				0.012
Yes	286	265 (92.7)	21 (7.3)	
No	139	118 (84.9)	21 (15.1)	
Consumption of flesh foods				<0.001
Yes	331	309 (93.4)	22 (6.6)	
No	94	74 (78.7)	20 (21.3)	

In multivariate analysis, sex of child, meeting the daily minimum dietary diversity and maternal body mass index were found associated with wasting in children. These variables in the model equation explained 13.4% of the variance (Nagelkerke R Square = 0.134) in wasting. Compared to female children, male children were 2.4 times more likely to experience wasting [AOR = 2.40; 95% CI (1.189–4.844); *p* = 0.015]. The odds of wasting was higher among children who did not meet the minimum dietary diversity. Compared to children who met the minimum dietary diversity, children who did not meet it were 3.7 times more likely to be wasted [(AOR = 3.733; 95% CI (1.889–7.376); *p* < 0.001]. The odds of child wasting was significantly higher among underweight mothers [AOR = 3.897; 95% CI (1.404–10.820); *p* = 0.009] compared to mothers who had normal body mass index (Table 9).

**Table 9:** Multivariate analysis of the determinants of wasting in children

	*p* value	AOR	95% C.I. for AOR	
			Lower	Upper
Sex of child				
Female			Reference	
Male	0.015	2.400	1.189	4.844
Minimum dietary diversity				
Yes			Reference	
No	<0.001	3.733	1.889	7.376
BMI of mother	0.032			
Normal			Reference	
Underweight	0.009	3.897	1.404	10.820
Overweight	0.811	1.109	.474	2.597
Constant	0.000	.030		

### Predictors of underweight among children

In bivariate analysis, sex of child, birth weight, maternal body mass index, possession of toilet facility predicted underweight in children. Children who lived in households with a toilet facility were less likely to be underweight. Underweight mothers were also more likely to have an underweight child (Table 10).

**Table 10:** Bivariate analysis of predictors of underweight among children

Characteristic	N	Classification of underweight		*p* value
		Normal	Underweight	
Sex				0.005
Male	216	163 (75.5)	53 (24.5)	
Female	209	180 (86.1)	29 (13.9)	
Birth weight				0.008
< 2.5 Kg	23	14 (60.9)	9 (39.1)	
≥ 2.5 Kg	170	145 (85.3)	25 (14.7)	
BMI of mother				0.009
Underweight	27	19 (70.4)	8 (29.6)	
Normal	306	240 (78.4)	66 (21.6)	
Overweight	92	84 (91.3)	8 (8.7)	
Toilet				0.041
Pit latrine/flush	48	44 (91.7)	4 (8.3)	
No facility	377	299 (79.3)	78 (20.7)	

Only two of the predictors of underweight significant in bivariate analysis were able to reach significance in multivariate analysis. Sex of child and birth weight were associated with child underweight in multivariate analysis. These factors accounted for 10.9% of the variability (Nagelkerke R Square = 0.109) in underweight in children. Implying that other factors not in the equation, probably not measured in this study also explain underweight in children. Male children were 2.7 times more likely to be underweight [AOR = 2.685; 95% CI (1.205–5.98); *p* = 0.016] compared to female children. The odds of underweight was significantly higher among children who were born low birth weight than those born of adequate birth weight. Compared to children born of adequate birth weight, children born low birth weight were 3.8 times more likely to be underweight [AOR = 3.778; 95% CI (1.440–9.911); *p* < 0.001]. Table 11 presents the results of multivariate analysis of the determinants of underweight in children in the study sample.

**Table 11:** Multivariate analysis of the determinants of underweight in children

	*p* value	AOR	95% C.I. for AOR	
			Lower	Upper
Sex				
Female			Reference	
Male	0.016	2.685	1.205	5.981
Birth weight				
≥ 2.5 Kg			Reference	
< 2.5 Kg	0.007	3.778	1.440	9.911
Constant	0.000	.099		

## Discussion

This study sought to investigate the maternal and child factors that determine child stunting, wasting and underweight among children aged 6–59 months in Northern Ghana. The main findings were that maternal height associated negatively with stunting but not wasting. The factors that are associated with low height- for-age z-score may not necessarily be the same as stunting. In addition, infant and child feeding practices as measured by dietary diversity score associated positively with weight-for-height Z-scores than length-for-age Z-scores of young children and the consumption of some specific food groups including, animal source foods and legumes and eggs were associated with lower HAZ but with increased likelihood of higher WHZ among children. Stunting was associated with sex, maternal height and age of child, whereas wasting was associated with dietary diversity, sex and body mass index of mother. Child underweight was associated with sex and birth weight of children.

The prevalence of child malnutrition indicators measured in present study were lower than the regional prevalence of 33.1, 20.0 and 6.3% for stunting, underweight and wasting respectively in the recent Ghana demographic and health survey report [7], except for wasting where children in the study sample recorded slightly higher prevalence (9.9% versus 6.3% respectively). This implies that, though stunting and underweight are decreasing in the region, household food security does not seem to improve, as reflected in the higher prevalence of wasting found in present study. The lower prevalence of stunting and underweight recorded in this study compared to those reported in the 2014 demographic and health survey has also been documented by recent studies in the Northern region [9, 22].

A higher proportion of children in the study sample consumed a diversified diet from at least four food groups. Nearly sixty- four percent of the children met the minimum dietary diversity recommendation with an average dietary diversity score of 3.6. A study among children under five years in the Central region of Ghana however reported lower minimum dietary diversity scores [23]. Dietary diversity score was associated with higher mean WHZ and lower HAZ of children. This finding suggests that, increasing the diversity of child diet may have greater and immediate impact on WHZ and subsequently wasting. As dietary diversity is measured by 24 h recall, its negative association with HAZ suggests that there was a recent increase in dietary diversity of children, particularly stunted children which was unlikely to have an immediate effect on HAZ as stunting is a result of long term dietary inadequacy. This finding implies that, increasing dietary diversity of children, particularly those experiencing stunting may not lead to immediate improvements in HAZ. Surprisingly, the consumption of specific food groups including staples and eggs associated positively with WHZ but were negatively associated with HAZ. This further imply that, the infant feeding indicators relate better to WHZ than HAZ.

The association between older age and stunting is consistent with data from Ethiopia [24] and Bangladesh [25] which showed increase in stunting levels with increasing child age starting from first year and peaking at three years. Present study results show that, male children were 9.8 times more likely to experience stunting. This may be due to inadequate complementary feeding and repeated infections exposed to children in this age group. As these children gradually become less dependent on breast milk and become increasingly reliant on complementary foods, infections are more likely to occur especially in households where hygiene and sanitary practices are poor. Male children were also more likely to be stunted compared to female children. This relationship has been reported in the literature from developing countries including Ghana [14, 26], Tanzania [27], Ethiopia [24], Cambodia [3] and India [28]. In spite of this widespread identification of the male susceptibility to stunting, the exact factors underlying it have yet to be well understood [14] but likely to be biological [29]. Short maternal stature was associated with higher odds of child stunting in present study. This relationship has been reported by earlier studies. For example, in an analysis of data of 5 birth cohorts, it was established that short mothers were more likely to have a stunted child [30]. Data from other studies from countries of varying economic development have also shown this relationship [31–33]. The relationship may be explained by the intergenerational cycle of malnutrition [34], where stunted children grow to become stunted mothers who give birth to children who also become stunted. Present study results also show that being wasted, higher maternal height, protected drinking water source and introduction of complementary foods at 6 months were associated with positive HAZ, while being a wasted child, continued breastfeeding and increased age of child were associated negatively with HAZ. The results show that the factors that affect HAZ may not necessarily be the same as stunting.

Wasting was associated with sex of child, minimum dietary diversity and maternal body mass index. Male children had higher odds of acute malnutrition. This finding is consistent with earlier studies among preschool children [35, 36]. Children who did not meet the minimum dietary diversity were more likely to experience wasting. Wasting reflect current nutritional inadequacy therefore it is understandable that the consumption of at least four food groups in the previous day was associated with less risk of wasting. The association between maternal body mass index and wasting in children has been reported in Bangladesh [37]. Wasting decreased with increasing body mass index of child. This suggests that in households where there is enough food for mothers to maintain optimal nutritional status, there is equally enough for adequate child nutrition.

Underweight was high in the study sample, however only two factors were associated with it in multivariate analysis. These factors however explained an appreciable amount (10.9%) of the variance in underweight among children in the study sample. Obviously no single study can identify all the factors related to malnutrition considering the different contexts within which children find themselves. Sex and birth weight of child were associated with underweight in children. Male children were as well more likely to experience underweight than their female counterparts. The high likelihood of underweight among male children has been reported earlier [35]. Children born of adequate weight were less likely to be underweight than those who were born with low birth weight. This finding is consistent with an earlier study in Bangladesh [38]. This finding suggests that, nutritional well-being during pregnancy which is translated into adequate birth weight is important and may be protective against subsequent malnutrition.

This study is limited by the cross-sectional nature of the data, therefore causal conclusions were not possible. Our study has however, thrown more light on the maternal and child factors that determine undernutrition in children.

## Conclusion

Maternal height associated negatively with stunting but not wasting. Factors that affect low height –for-age z-score (HAZ) may not necessarily be the same as stunting. Infant and child feeding practices as measured by dietary diversity score associated positively with weight-for-height Z-scores than length-for-age Z-scores of young children in rural Northern Ghana. Surprisingly, consumption of some specific food groups including, animal source foods, legumes, staples and eggs were associated with lower HAZ but with increased likelihood of higher WHZ among children 6–59 months.

## Additional file

Additional file 1: Study questionnaire. (DOCX 28 kb)

## Abbreviations

ANC: Antenatal care
ANOVA: Analysis of variance
AOR: Adjusted odds ratio
CI: Confidence interval
HAZ: Height-for-age Z-score
SD: Standard deviation
WAZ: Weight-for-age Z-score
WHO: World Health Organization
WHZ: Weight-for-height Z-score
