# Supplementary material for: The effect of maternal and child factors on stunting, wasting and underweight among preschool children in Northern Ghana
Source: BMC Nutr. 2017 Apr 4;3:31. doi: 10.1186/s40795-017-0154-2 (PMC7050753; doi:10.1186/s40795-017-0154-2)
Supplement: Supplementary file 1 — Study questionnaire. (DOCX 28 kb) [file 40795_2017_154_MOESM1_ESM.docx]

**QUESTIONNAIRE**

**INFORMED CONSENT**

Good morning / afternoon / evening. I am a nutrition researcher from the University for Development Studies. I am conducting a study on (topic and explanation of measurements). I would like to have an interview with you on the topic and would very much appreciate your participation in this study. You and your child have been selected to be part of the study to respond to a questionnaire which will take about 30 minutes of your time. All of the answers you will give will be confidential and no one can trace them to you. If I should come to any question you don’t want to answer, just let me know and I will go on to the next question.

Your participation in the study is purely voluntary and so you are at liberty to opt out. We would however be grateful if you agree to participate since your views are important. May I now ask that you and your (NAME OF CHILD) participate in the interview?

Now please tell me if you agree to take part in the study.

Agreed: (sign or thumb print) ----------------------------------------------------

Declined: (tick) ---------------------------------------------------------------------- [ ]

**IDENTIFICATION**

1. Date of interview: ͟ ͟ / ͟ ͟ / 2016 (dd/mm/yyyy)

1. Name of Region ………………………………………………………
2. District Name …………………………………………………………..
3. Sub-district Name ………………………………………………………
4. Cluster Name ……………………………………………………………
5. Household Name ……………………………………………………….
6. Child’s Name ……………………………………………………………
7. Questionnaire No ……………………………………………………….

**SECTION A: SOCIO-DEMOGRAPHIC CHARACTERISTICS OF SAMPLE**

**(Administer to the mother / Caregiver with children 6-59 months)**

1. Age of mother/caregiver …………………………………………………(years)
2. What is your Religion?
3. Christianity
4. Islam
5. ATR
6. Others (specify):
7. Marital Status
8. Single
9. Married
10. Divorced
11. Widow
12. Separated
13. Others (specify) …………………………………
14. Ethnicity of respondent?

1. Gonja

2. Dagomba

3. Sissala

4. Eve

5. Other (specify)…………………………………..

1. Aside from your own housework what do you do to earn income?
2. Trader/vendor
3. Agricultural worker (e.g. farmer)
4. Office worker (Civil Servant)
5. Service worker (e.g. Hair-dresser, seamstress)
6. Education/research (e.g. Teacher)
7. Healthcare (e.g. Nurse)
8. Nothing
9. Others (specify):
10. Mother’s highest educational level completed:
11. None
12. Primary
13. Middle/J.H.S
14. S.H.S/Vocational training
15. Tertiary
16. Others (specify)
17. How many children under five years of age live in your household? ………………..

**SECTION B: PAST OBSTETRIC DATA AND MEDICAL HISTORY**

1. Number of pregnancies**----------------------------------------------------------------**

2. Number of live deliveries**--------------------------------------------------------------**

3. At what gestational age (months of pregnancy) did you start the antenatal clinic visits?.......................................

4. Have you had any problems with this pregnancy? [1] Yes [2] No

5. If yes what was/were the problem(s)

[1] Bleeding [2] dizziness [3] abdominal pains [4] waist pains

[5] Headache [6] swollen feet [7] others (specify) ………………………..

6 Record from the mother’s antenatal card the number of times she visited a health care

center for prenatal care services during pregnancy with [child’s name]……………………

7. Where did you deliver (Name of child)?

1. At home
2. CHPS Compound
3. Clinic
4. Maternity home
5. Health centre
6. Hospital
7. Traditional Birth Attendant

**SECTION C: HEALTH STATUS ASSESSMENT**

1. Blood pressure at recruitment (first trimester)……………………….

2. Blood pressure at 36 weeks gestation………………………………..

3. Number of Malarial infections during last pregnancy…………………….

4. Had candidiasis during pregnancy? ......................................................

5. Complete the table below for maternal Hb during the pregnancy with (Child’s Name) using mother’s ANC book

| **Stage of pregnancy** | **Haemoglobin level in g/dl** |
| --- | --- |
| First trimester |  |
| Second trimester |  |
| Third trimester |  |

**SECTION D: DIETARY INTAKE OF CHILD**

1. Yesterday did [child’s name] eat any solid or semi-solid foods?

1. Yes
2. No

2**.** How many times did (Name of child) eat solid or semi-solid food or soft foods other than liquids yesterday during the day or at night? …………………………….

3a Please, mention all the foods and drinks that were eaten by (Name of child) over the past 24 hours whether at home or outside the home. (Hint: start with meal eaten at supper yesterday).

| Eating moment | Name of dish | Ingredients |
| --- | --- | --- |
| Breakfast |  |  |
| Snack before lunch |  |  |
| Lunch |  |  |
| Snack before dinner |  |  |
| Dinner |  |  |
| Snack after dinner |  |  |
| Drinks |  |  |

3. b. From the meals mentioned by the mother, indicate whether (Name of child), ate from the following food groups during the past 24hours whether at home or outside the home. (YES=1, NO=0)

| Food group | Examples | YES | NO |
| --- | --- | --- | --- |
| Grain, roots and tubers | Cereals, White tubers and roots |  |  |
| Dairy products | Milk and milk products |  |  |
| Flesh foods | Organ meat, Flesh meats and Fish |  |  |
| Eggs | Eggs |  |  |
| Legumes | Legumes, nuts and seeds |  |  |
| Vitamin A- rich fruits and vegetables | Dark green leafy vegetables,Fresh vitamin A-rich fruits, Vitamin A-rich vegetables and tuber and Oils and fats |  |  |
| Other fruits and vegetables | Dried fruits and vegetables |  |  |

**SECTION E:** **INFANT AND YOUNG CHILD FEEDING (IYCF) PRACTICES**

1. After delivery of (Name of child), how long did it take you to breastfeed him/her for the first time?

1. Within first hour of delivery
2. 2 to 23 hours after delivery
3. The next day (More than 24 hours)
4. Do not remember

2. Before putting (Name of child) to the breast for the first time after delivery, what was child given to drink? (Multiple responses possible)

1. Nothing
2. Milk (other than breast milk)
3. Plain water
4. Sugar or glucose water
5. Gripe water
6. Sugar-salt-water solution
7. Fruit juice
8. Infant formula
9. Tea / coffee
10. Honey
11. Other (specify) _____________

3. When you delivered (Name of child) what did you do with the first yellowish breast milk?

(1) Gave it to the baby (2) Discarded it (3) Other (Specify) _____________

4. Is (child’s name) currently breastfeeding?

(1) Yes (2) No

5. Yesterday did [child’s name] have anything to drink from a bottle with a nipple during the day or night? (1) Yes (2) No

6. Is child currently eating other foods apart from breast milk? (1) Yes (2) No

7. At what age did you first give solid or semisolid food to [child’s name]?

1. Before 6 months
2. At Six months
3. Seven to 9 months
4. After nine months
5. Yet to start
6. Don’t know

**SECTION F: CHILD MORBIDITY AND UTILIZATION OF HEALTH SERVICES**

1. Has (Name of child) had an illness with a cough that comes from the chest at any time in the last two weeks? (1).Yes (2). No (3). Don’t know

2. Did (Name of child) get diarrhoea in the past two weeks? (Diarrhoea is having loose watery stools more than 3 times). (1). Yes (2). No (3). Don’t know

3. Has (Name of child) had Fever/Malaria: High temperature with shivering/ suspected malaria in the last two weeks? (1) Yes (2) No (3) Don’t know

**SECTION G: WATER, SANITATION AND HYGIENE**

1. What is the main source of drinking water for members of your household? (Only one response)
2. Piped water
3. Borehole
4. Protected well
5. Unprotected well
6. Surface water ( river, stream, dam, lake, pond, canal, irrigation channel)
7. Rain water
8. Other (Specify) …………………………………………………………………..
9. Are you satisfied with the drinking water supply in this community? (IF ANSWER IS 2,3 OR 4 GO TO question 4)
10. Yes
11. No
12. Partially
13. Don’t know
14. What is the main reason you are not satisfied with the water supply?
15. Not enough
16. Long waiting queue
17. Long distance
18. Irregular supply
19. Bad taste
20. Water too warm
21. Bad quality
22. Have to pay
23. Other (specify)
24. Don’t know
25. What kind of toilet facility does this household use?
26. Flushed type
27. Simple pit latrine with floor/slab
28. Pit latrine without floor/slab
29. No facility, field, bush, plastic bag
30. Do you have children under three years old? ( IF ANSWER IS 2 GO TO question 7)
31. Yes
32. No
33. The last time (Name of Youngest Child) passed stools, what was done to dispose of the stools?
34. Child used toilet/latrine
35. Put/rinsed into toilet or latrine
36. Buried
37. Thrown into garbage
38. Put/rinsed into drain or ditch
39. Left in the open
40. Other
41. Don’t know
42. At what moments did you wash your hands in the last 24 hours? (Multiple answers possible) (Probe; “Any other times?”)
43. Before preparation of food
44. After going to toilet
45. Before eating food
46. After eating food
47. Before feeding a child
48. Other (Specify) …………………………………………..

**SECTION H: SOCIO-ECONOMIC STATUS ASSESSMENT**

1. What type of house do members of the household dwell in?
2. Block house (2) Brick house (3) Mud house (4) Others (specify) ………
3. What kind of toilet facility do members of the household usually use?
4. Own flush toilet
5. Public or shared flush toilet
6. Own pit toilet
7. Public or shared pit toilet
8. No facility (bush)
9. What is the main source of lighting for the household?
10. Electricity (2) Solar (3) Kerosene (4) Others (specify) ……..……
11. What type of fuel does your household mainly use for cooking?
12. Electricity
13. LPG
14. Charcoal
15. Kerosene
16. Firewood
17. Others (specify) ……………………………………
18. Does your household have any of these assets? (Tick Yes(1) or No(0))

| ITEM | YES | NO |
| --- | --- | --- |
| Radio |  |  |
| Color/black TV |  |  |
| Satellite dish |  |  |
| Sewing Machine |  |  |
| Mattress |  |  |
| Refrigerator |  |  |
| DVD/VCD |  |  |
| Computer |  |  |
| Electric Fan |  |  |
| Mobile Telephone |  |  |
| Bicycle |  |  |
| Motorcycle/Tricycle |  |  |
| Animal-drawn cart |  |  |
| Car/Truck |  |  |

**SECTION I: ANTHROPOMETRY MEASUREMENT (MOTHER)**

Height: ……………………………..cm

Weight: ……………………………..kg

BMI...................................................kg/m^2^

Gestational age at delivery…………………………………… (Completed weeks)

**SECTION J: INFANT ANTHROPOMETRY**

Sex of child: (1). Male (2). Female

Date of birth: ___/___/____ (dd/mm/yyyy)

Date of birth verified from:

1. Birth certificate
2. Health records booklet
3. Community register
4. Other document (specify)…………………..
5. Could not verify

Age of child (months):____________

Baby’s birth weight (record from child health records booklet)…………………… (kg)

Weight of child: __ __.__ (kg)

Height of child: __ __ __ .__ (cm)

Presence of bilateral pitting oedema? (1) Yes (2). No

**THANK YOU, END OF INTERVIEW**
